# Interventional Cardiology: Current Challenges in Acute Myocardial Infarction

**DOI:** 10.3390/jcm11154504

**Published:** 2022-08-02

**Authors:** Andreas Schäfer

**Affiliations:** Department of Cardiology and Angiology, Hannover Medical School, Carl-Neuberg-Str. 1, 30625 Hannover, Germany; schaefer.andreas@mh-hannover.de; Tel.: +49-511-532-5240

Welcome to the Special Issue “Interventional cardiology: current challenges in acute myocardial infarction” in the *Journal of Clinical Medicine*. This issue is dedicated to contemporary challenges in cardiogenic shock as well as the reduction in infarct size due to limiting reperfusion injury in patients with acute myocardial infarction (AMI) undergoing primary percutaneous coronary intervention (pPCI). This issue focuses on these topics, which are either innovative in the field of reperfusion injury or highly debated in the area of cardiogenic shock. The Special Issue appears in an open access journal dedicated to fostering worldwide dissemination of these emerging issues in AMI care.

Recent early pharmacological interventions for acute myocardial infarction have focused primarily on lipid-lowering therapy and anti-platelet treatments. With regard to lipid lowering, recent guidelines set lower values than previous targets for LDL cholesterol [1], and current strategies aim for very early combination therapies to address these lower thresholds [2,3]. Clearly stratified protocols enable patients to reach those ambitious targets more often than historically observed [4]. Anti-platelet treatment before, during and after pPCI has been simplified [5], but the approach of abandoning oral pre-treatment with P2Y12 inhibitors, with a preference for prescribing prasugrel over ticagrelor, has been intensely debated [6,7]. However, in the current issue, we discuss interventions prior to or during pPCI aiming to reduce early or chronic heart failure in those patients.

AMI is among the major contributors to cardiovascular morbidity and mortality. In the acute phase, cardiogenic shock (CS) critically contributes to in-hospital mortality [8]. Recommending complete revascularisation in AMI-CS patients was a long-lasting standard in previous guidelines, until the results of the prospective CULPRIT-SHOCK trial was published [9], completely changing the view on revascularisation in AMI-CS patients. However, one still-unresolved issue regards the use of mechanical circulatory support (MCS) in AMI-CS patients, which was only used in the minority of patients in CULPRIT-SHOCK. Retrospective analyses had reported an association of complete revascularisation on MCS devices with reduced mortality [10,11]. In this issue of the *Journal of Clinical Medicine*, Masiero et al. discuss the role of different MCS strategies in optimal revascularisation in patients with AMI-CS [12].

Regarding MCS in AMI-CS patients, its potential beneficial influence still remains to be clarified. While many retrospective analyses have suggested a beneficial influence of micro-axillary flow pumps [13,14] and/or extracorporeal membrane oxygenation (ECMO) [15,16] on mortality in those patients, several clinical trials, such as DanGer [17], ECLS-SHOCK [18] and EURO-SHOCK [19], have not yet concluded. In this issue, Møller et al. discuss the role of the Impella device [20], whereas Freund et al. discuss the role of ECMO in AMI-CS patients [21].

While AMI-CS threatens the patient’s life during the acute hospitalisation for AMI, rehospitalisation for acute heart failure is a major contributor to morbidity following the acute phase. Meta-analyses have shown that infarct size is strongly associated with all-cause mortality and hospitalisation for heart failure within 1 year [22]. This finding resulted in infarct size gaining more promise as a surrogate parameter of adverse clinical outcomes. Hence, several interventions hoping to reduce infarct size following acute STEMI have been recently described, because animal data suggest that almost half of the final infarct size might be attributable to the phenomenon of reperfusion injury [23,24]. Two tempting approaches to reduce infarct size in addition to rapidly performing pPCI have recently been described. While a pilot trial rapidly applying systemic hypothermia to reduce infarct size did not provide positive conclusive results [25], selective intracoronary cooling seems to be a safe and promising approach [26]. In this issue, El Farissi et al. discuss the role of intraprocedural hypothermia during reperfusion [27]. The other technique aiming to mitigate reperfusion injury is intracoronary application of super-saturated oxygen, which allows for diffusion of dissolved oxygen into the periphery of the ischaemic area and reduces microvascular obstruction by preventing endothelial cell edema [28]. This approach does not delay state-of-the art revascularisation and can be initiated within five minutes after the end of pPCI. Earlier studies demonstrated an approximate absolute 6–7% reduction in infarct size in anterior STEMI, which translates into an almost 20% reduction of 1-year all-cause mortality and heart failure hospitalisation [22,29]. In this issue, Schäfer et al. discuss the role of intracoronary application of super-saturated oxygen for infarct size reduction [30].

In this issue of the *Journal of Clinical Medicine*, clinical scientists report their recent advances in this field. They have all not only performed proof-of-principle investigations but also prospective clinical trials to ensure that the data provided are credible and accountable. Once the respective trials are fully published, we will evaluate whether those promising approaches can translate into standard of care and help to improve patient outcomes. Let us all work together to prevent acute and chronic heart failure in AMI patients, because—as stated by the Apollo astronauts decades ago—failure is not an option.
